# Editorial: Innovative approaches to nutrition counseling in pediatric dietetics - guidelines, practices, and future directions

**DOI:** 10.3389/fnut.2026.1827077

**Published:** 2026-04-14

**Authors:** Karolina Krupa-Kotara, Agnieszka Kozioł-Kozakowska, Michał Brzeziński

**Affiliations:** 1 Department of Epidemiology, Faculty of Public Health in Bytom, Medical University of Silesia, Katowice, Poland; 2 Laboratory of Pediatric Dietetics, Department of Pediatric Gastroenterology and Nutrition, Jagiellonian University, Cracow, Poland; 3 Departments and Clinics of Pediatrics, Gastroenterology, Allergology, and Child Nutrition, Medical University of Gdańsk, Gdańsk, Poland

**Keywords:** dietetic interventions, family-centered care, pediatric nutrition counseling, personalized nutrition, public health nutrition

## 1 Introduction

Nutrition counseling in pediatric dietetics represents a cornerstone of health promotion and the prevention and management of developmental disorders and diseases. It encompasses not only supporting optimal growth and development but also preventing and treating eating disorders, obesity, undernutrition, and chronic conditions. Over recent decades, substantial advances have been made in understanding children's nutritional requirements, metabolic pathways, the role of the gut microbiome, and the influence of environmental and psychosocial factors on eating behaviors. At the same time, clinical practice increasingly demonstrates that the effectiveness of nutritional interventions in pediatrics depends not solely on the content of dietary recommendations, but equally on the quality of the counseling process, continuity of care, and the capacity to adapt recommendations to the individual context of the child and family.

Despite the availability of increasingly detailed clinical guidelines, pediatric nutrition counseling remains an area characterized by marked heterogeneity of practice, both across countries and within healthcare systems. This variability extends to documentation of the nutrition care process, approaches to infant feeding, the extent to which psychosocial determinants are addressed, and the use of emerging technologies for monitoring and patient support. These challenges are further compounded by the growing complexity of pediatric health needs, as children increasingly live in environments shaped by social inequalities, limited access to resources, caregiver stress, and mental health burdens.

Against this backdrop, there is growing recognition of the need to move beyond models of nutrition counseling based on isolated consultations toward process-oriented, interdisciplinary, and longitudinal approaches. Contemporary pediatric dietetics requires integrating clinical and biological data with insights into health behaviors, family dynamics, and environmental determinants. In parallel, the rapid development of digital health tools and telemedicine offers new opportunities to enhance continuity of care, particularly for children with chronic conditions, disabilities, or restricted access to specialized services.

**Figure 1:**
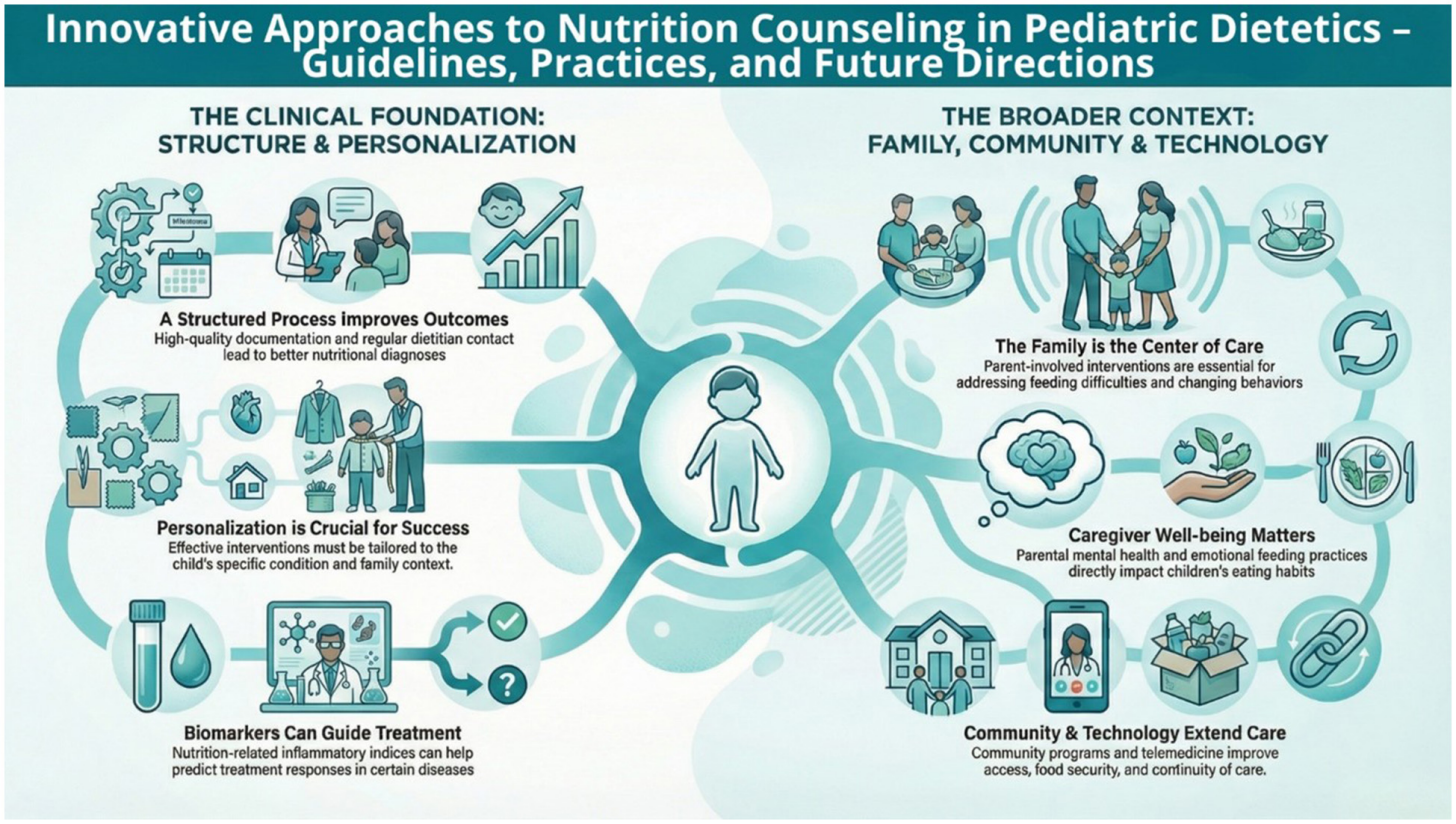
Conceptual model of innovative approaches to nutrition counseling in pediatric dietetics, including clinical foundations, family-centered care, community support, and technology-assisted care.

This Research Topic aimed to bring together and synthesize current evidence on innovative approaches to nutrition counseling in pediatric dietetics, encompassing clinical guidelines, therapeutic practice, and future directions for the field. The collected contributions conceptualize pediatric nutrition counseling as a multidimensional process that incorporates standardized care pathways, individualized interventions, family and environmental contexts, biomarkers, and technological solutions. The overarching objective was to identify models and strategies to improve the quality, effectiveness, and accessibility of nutrition counseling for children and their families across diverse clinical and social settings. The conceptual framework of innovative approaches to nutrition counseling in pediatric dietetics, including clinical, family, community, and technology-related components, is presented in Figure 1.

## 2 Quality, structure, and standardization of nutrition counseling

A prominent theme within this Research Topic is the importance of a well-structured nutrition care process. Gaubert et al. demonstrate that higher-quality documentation of the Nutrition Care Process and more frequent dietitian contact are associated with improved nutrition diagnoses in breastfed infants. These findings highlight that innovation in nutrition counseling begins with robust organizational and quality foundations.

A similar need for coherence is underscored by Karabayir et al., who report substantial variability in recommendations for complementary feeding, influenced by physician experience and characteristics. Their results emphasize the necessity of standardized clinical education and harmonized practices during this critical period of infant nutrition.

The scoping review by Neri et al. evidence on prenatal and postnatal nutrition counseling interventions designed to support breastfeeding. The authors show that structured counseling, delivered both in person and remotely, is associated with higher exclusive breastfeeding rates, longer breastfeeding duration, and improved maternal self-efficacy. The review highlights mobile-based and peer-support interventions as particularly promising approaches, particularly in low-resource settings, and underscores the effectiveness of targeted nutrition counseling as a component of breastfeeding support.

## 3 Family and psychosocial determinants of eating behaviors

Several contributions highlight that effective pediatric nutrition counseling cannot be separated from the family context. da Fonseca et al. show that parent-involved interventions are essential in addressing feeding difficulties in both neurotypical and neurodivergent children, underscoring the importance of shared behavioral and educational strategies.

From a public health perspective, Sarkkola et al. demonstrate that parental depression and emotional feeding practices are independently associated with a tendency toward overeating in preadolescents. These findings reinforce the view that caregiver mental health is a critical component of effective nutrition counseling.

## 4 Personalization of nutrition in pediatric disorders and diseases

Personalized nutrition interventions in chronic and rare pediatric conditions constitute another key pillar of this Research Topic. Yilmaz Nas et al. show that successful transitions between protein substitutes in children with phenylketonuria depend not only on metabolic control, but also on caregiver education and school-based support.

In the context of acute inflammatory disease, Yi et al. demonstrate that nutrition-related inflammatory indices can predict treatment response in Kawasaki disease, highlighting the growing role of nutritional biomarkers in clinical decision-making.

## 5 Nutrition, the gut microbiome, and neurodevelopment

An emerging direction for future research is articulated in the review by Jiang and Li, which synthesizes evidence linking early-life nutrition and gut microbiota composition to brain development and behavior. This work underscores the potential of dietary interventions as tools to support neurocognitive development.

## 6 Population- and environment-based interventions—From undernutrition to obesity

A substantial body of work emphasizes that pediatric nutrition counseling must be embedded within broader population and environmental contexts. Kim et al. identify lifestyle factors such as sleep duration, physical activity, and meal regularity as key correlates of adolescent obesity.

Similarly, Halilagic et al. show that effective interventions for children in socially disadvantaged circumstances require multilevel, long-term strategies.

This perspective is further enriched by Defeyter et al., who demonstrate that community-based programs can enhance physical activity, household food security, and caregiver wellbeing.

At the same time, Liu et al. illustrate that long-term growth monitoring and nutrition promotion programs can effectively address undernutrition in low-resource settings, underscoring the global dimension of pediatric nutrition counseling.

## 7 Technology and emerging models of care

New models of care are represented by the review of Tagi et al., which shows that telemedicine can substantially improve continuity of nutritional care and quality of life for children with severe neurological impairment and their families.

## 8 Conclusions and future directions

Collectively, the articles in this Research Topic indicate that pediatric nutrition counseling should be conceptualized as a complex, multidimensional, and longitudinal process that requires the integration of clinical, biological, and psychosocial knowledge. The evidence highlights the importance of standardized care processes to enhance diagnostic accuracy and outcome monitoring, as well as the need for individualized dietary recommendations that account for both biological parameters and family-level determinants.

A consistent finding across studies is the central role of the family and immediate environment in shaping sustainable dietary behavior change in children. Effective nutrition counseling, therefore, necessitates active caregiver involvement and careful consideration of home and community contexts. In addition, population-based and community-anchored interventions demonstrate considerable potential to support public health objectives, addressing both obesity prevention and undernutrition.

Finally, the integration of psychosocial factors, caregiver mental health, and systematic monitoring of child development emerges as a key determinant of counseling effectiveness. Taken together, these contributions delineate a coherent landscape of current directions in pediatric dietetics and underscore the need for continued development of interdisciplinary care models that combine clinical tools, family support, and population-level approaches to improve the quality and impact of pediatric nutrition counseling.

